# Bis{1-[(*o*-tol­yl)imino­meth­yl]-2-naphthol­ato}copper(II)

**DOI:** 10.1107/S160053681003374X

**Published:** 2010-08-28

**Authors:** Peihua Zhu, Jiemei Yu, Hongyan Wang, Chunlai Zhang, Qin Wei

**Affiliations:** a School of Chemistry and Chemical Engineering, University of Jinan, Jinan 250022, People’s Republic of China;

## Abstract

In the title complex, [Cu(C_18_H_14_NO)_2_], the Cu^II^ ion lies on a crystallographic inversion centre and is bonded to the *O*- and *N*-donor atoms of the two bidentate chelate 1-[(*o*-tol­yl)imino­meth­yl]-2-naphtho­late ligands in a *trans* arrangement. The distorted square-planar geometry about Cu^II^ has normal dimensions, with Cu—O = 1.8881 (15) Å and Cu—N = 1.9804 (17) Å.

## Related literature

For general background to Schiff base complexes of copper(II), see: Adsule *et al.* (2006 ▶); Barton & Ollis (1979 ▶); Layer (1963 ▶); Ingold (1969 ▶); Erxleben & Schumacher (2001 ▶). For related structures, see: Kaitner *et al.* (1998 ▶).
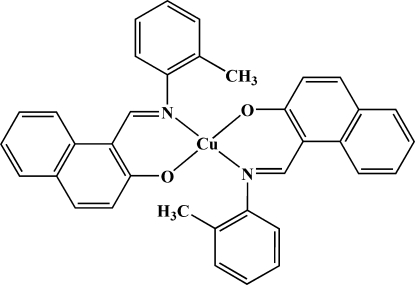


## Experimental

### 

#### Crystal data

[Cu(C_18_H_14_NO)_2_]
*M* *_r_* = 584.14
Monoclinic, 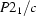

*a* = 7.39342 (16) Å
*b* = 22.0666 (5) Å
*c* = 8.74653 (19) Å
β = 95.775 (2)°
*V* = 1419.73 (5) Å^3^
*Z* = 2
Mo *K*α radiation
μ = 0.81 mm^−1^
*T* = 293 K
0.19 × 0.15 × 0.14 mm

#### Data collection

Bruker APEXII CCD area-detector diffractometer
Absorption correction: multi-scan (*SADABS*; Sheldrick, 2003 ▶) *T*_min_ = 0.862, *T*_max_ = 0.896
7361 measured reflections
2882 independent reflections
2058 reflections with *I* > 2σ(*I*)
*R*_int_ = 0.023

#### Refinement

*R*[*F* ^2^ > 2σ(*F* ^2^)] = 0.037
*wR*(*F* ^2^) = 0.098
*S* = 1.05
2882 reflections
158 parameters
H-atom parameters constrained
Δρ_max_ = 0.57 e Å^−3^
Δρ_min_ = −1.04 e Å^−3^

### 

Data collection: *APEX2* (Bruker, 2004 ▶); cell refinement: *SAINT-Plus* (Bruker, 2001 ▶); data reduction: *SAINT-Plus*; program(s) used to solve structure: *SHELXS97* (Sheldrick, 2008 ▶); program(s) used to refine structure: *SHELXL97* (Sheldrick, 2008 ▶); molecular graphics: *SHELXTL* (Sheldrick, 2008 ▶); software used to prepare material for publication: *SHELXTL*.

## Supplementary Material

Crystal structure: contains datablocks global, I. DOI: 10.1107/S160053681003374X/zs2057sup1.cif

Structure factors: contains datablocks I. DOI: 10.1107/S160053681003374X/zs2057Isup2.hkl

Additional supplementary materials:  crystallographic information; 3D view; checkCIF report

## Figures and Tables

**Fig. 1.**
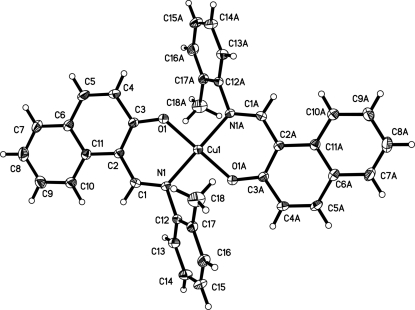
The molecular structure and atom numbering scheme for (I), with 25% probability displacement ellipsoids for non-H atoms. The A atoms are related by crystallographic inversion symmetry (code: -x + 1, -y + 1, -z + 1).

## supplementary crystallographic information

### Comment

Schiff bases are used as starting materials in the synthesis of important drugs (Barton *et al.*, 1979; Layer, 1963; Ingold, 1969). For easy preparation and structural variation, a large number of complexes with schiff bases have been reported. The copper(II) complexes with schiff bases have been studied for their applications in the design and construction of new magnetic materials (Erxleben *et al.*, 2001) and for their cellular proteasome activity (Adsule *et al.*, 2006). We report here the crystal structure of the new copper(II) complex with the Schiff base ligand *N*-(*o*-tolyl)-2-hydroxy-1-naphthaldimine, the title compound [Cu(C_18_H_14_N O)_2_] (I).

The molecular structure of (I) is shown in Fig. 1. The Cu^II^ ion is four-coordinated with two O and two N donor atoms from two bidentate chelate Schiff base ligands, resulting in a square planar geometry. The discrete complex units have distorted square planar geometry with the Cu lying on a crystallographic inversion centre, the Cu1—O1 and Cu1—N1 bond lengths [1.8881 (15) and 1.9804 (17) Å respectively] being normal for this configuration. It is worth noting that the C1—N1—C12—C17 torsion angle is 55.5 (2)° in the title complex, while in the free ligand the angle is 34.1 (3)°, and this is ascribed to steric hindrance caused by the close approach in the title complex of the two ligands (Kaitner *et al.*, 1998). The adjacent complex molecules are stacked with no significant intermolecular interactions.

### Experimental

Copper(II) acetate hydrate (0.199 g, 0.001 mol) in methanol (50 ml) and *N*-(*o*-tolyl)-2-hydroxy-1-naphthaldimine (0.586 g, 0.002 mol) in acetonitrile (75 ml) were mixed and heated at 333 K for 1 h. The solution was filtered and the filtrate kept in a beaker at room temperature for crystallization. Black crystals of (I) started appearing after 3 days: yield, 0.667 g (85%).

### Refinement

Hydrogen atoms were placed in calculated positions and refined using a riding-model approximation with C—H = 0.93 Å, *U*_iso_ = 1.2*U*_eq_ (C) for aromatic H atoms and C—H = 0.96 Å, *U*_iso_ = 1.5*U*_eq_ (C) for methyl H atoms.

### Crystal data

**Table:**

[Cu(C_18_H_14_NO)_2_]	*F*(000) = 606
*M**_r_* = 584.14	*D*_x_ = 1.366 Mg m^−^^3^
Monoclinic, *P*2_1_/*c*	Mo *K*α radiation, λ = 0.71073 Å
Hall symbol: -P 2ybc	Cell parameters from 3786 reflections
*a* = 7.39342 (16) Å	θ = 2.9–28.9°
*b* = 22.0666 (5) Å	µ = 0.81 mm^−^^1^
*c* = 8.74653 (19) Å	*T* = 293 K
β = 95.775 (2)°	Block, red
*V* = 1419.73 (5) Å^3^	0.19 × 0.15 × 0.14 mm
*Z* = 2	

### Data collection

**Table:**

Bruker APEXII CCD area-detector diffractometer	2882 independent reflections
Radiation source: fine-focus sealed tube	2058 reflections with *I* > 2σ(*I*)
graphite	*R*_int_ = 0.023
φ and ω scans	θ_max_ = 26.4°, θ_min_ = 2.9°
Absorption correction: multi-scan (*SADABS*; Sheldrick, 2003)	*h* = −9→9
*T*_min_ = 0.862, *T*_max_ = 0.896	*k* = −27→26
7361 measured reflections	*l* = −10→10

### Refinement

**Table:**

Refinement on *F*^2^	Primary atom site location: structure-invariant direct methods
Least-squares matrix: full	Secondary atom site location: difference Fourier map
*R*[*F*^2^ > 2σ(*F*^2^)] = 0.037	Hydrogen site location: inferred from neighbouring sites
*wR*(*F*^2^) = 0.098	H-atom parameters constrained
*S* = 1.05	*w* = 1/[σ^2^(*F*_o_^2^) + (0.055*P*)^2^ + ] where *P* = (*F*_o_^2^ + 2*F*_c_^2^)/3
2882 reflections	(Δ/σ)_max_ < 0.001
158 parameters	Δρ_max_ = 0.57 e Å^−^^3^
0 restraints	Δρ_min_ = −1.04 e Å^−^^3^

### Special details

**Table:**

Geometry. All e.s.d.'s (except the e.s.d. in the dihedral angle between two l.s. planes) are estimated using the full covariance matrix. The cell e.s.d.'s are taken into account individually in the estimation of e.s.d.'s in distances, angles and torsion angles; correlations between e.s.d.'s in cell parameters are only used when they are defined by crystal symmetry. An approximate (isotropic) treatment of cell e.s.d.'s is used for estimating e.s.d.'s involving l.s. planes.
Refinement. Refinement of *F*^2^ against ALL reflections. The weighted *R*-factor *wR* and goodness of fit *S* are based on *F*^2^, conventional *R*-factors *R* are based on *F*, with *F* set to zero for negative *F*^2^. The threshold expression of *F*^2^ > σ(*F*^2^) is used only for calculating *R*-factors(gt) *etc*. and is not relevant to the choice of reflections for refinement. *R*-factors based on *F*^2^ are statistically about twice as large as those based on *F*, and *R*- factors based on ALL data will be even larger.

### Fractional atomic coordinates and isotropic or equivalent isotropic displacement parameters (Å^2^)

**Table:**

	*x*	*y*	*z*	*U*_iso_*/*U*_eq_	
C1	0.2037 (3)	0.50730 (10)	−0.2657 (3)	0.02945 (14)	
H1	0.2543	0.5301	−0.3401	0.035*	
C2	0.1929 (3)	0.44372 (10)	−0.2912 (3)	0.02945 (14)	
C3	0.1469 (3)	0.40463 (10)	−0.1721 (2)	0.02945 (14)	
C4	0.1653 (4)	0.34085 (11)	−0.1907 (3)	0.0421 (6)	
H4	0.1347	0.3151	−0.1132	0.051*	
C5	0.2260 (4)	0.31703 (11)	−0.3183 (3)	0.0435 (6)	
H5	0.2372	0.2752	−0.3259	0.052*	
C6	0.2735 (3)	0.35406 (11)	−0.4415 (3)	0.0356 (5)	
C7	0.3357 (4)	0.32855 (12)	−0.5738 (3)	0.0445 (6)	
H7	0.3507	0.2868	−0.5792	0.053*	
C8	0.3746 (4)	0.36357 (13)	−0.6944 (3)	0.0505 (7)	
H8	0.4166	0.3461	−0.7809	0.061*	
C9	0.3505 (4)	0.42579 (13)	−0.6864 (3)	0.0461 (7)	
H9	0.3744	0.4498	−0.7693	0.055*	
C10	0.2922 (3)	0.45262 (12)	−0.5584 (3)	0.0388 (6)	
H10	0.2783	0.4945	−0.5560	0.047*	
C11	0.2529 (3)	0.41766 (10)	−0.4298 (2)	0.0302 (5)	
C12	0.1859 (3)	0.60096 (10)	−0.1439 (3)	0.0303 (5)	
C13	0.1197 (3)	0.63778 (11)	−0.2651 (3)	0.0378 (6)	
H13	0.0541	0.6209	−0.3510	0.045*	
C14	0.1508 (4)	0.69953 (12)	−0.2586 (3)	0.0508 (7)	
H14	0.1067	0.7241	−0.3403	0.061*	
C15	0.2467 (4)	0.72443 (12)	−0.1319 (4)	0.0545 (8)	
H15	0.2672	0.7660	−0.1272	0.065*	
C16	0.3128 (4)	0.68777 (12)	−0.0114 (3)	0.0476 (7)	
H16	0.3790	0.7051	0.0736	0.057*	
C17	0.2832 (3)	0.62580 (10)	−0.0135 (3)	0.0351 (6)	
C18	0.3561 (4)	0.58665 (14)	0.1189 (3)	0.0566 (8)	
H18B	0.2568	0.5702	0.1683	0.085*	
H18C	0.4319	0.6106	0.1913	0.085*	
H18A	0.4263	0.5542	0.0819	0.085*	
Cu1	0.0000	0.5000	0.0000	0.02945 (14)	
N1	0.1508 (2)	0.53716 (8)	−0.1497 (2)	0.02945 (14)	
O1	0.0924 (2)	0.42288 (7)	−0.04412 (16)	0.02945 (14)	

### Atomic displacement parameters (Å^2^)

**Table:**

	*U*^11^	*U*^22^	*U*^33^	*U*^12^	*U*^13^	*U*^23^
C1	0.0365 (2)	0.0225 (2)	0.0305 (2)	−0.00170 (16)	0.00937 (14)	0.00038 (15)
C2	0.0365 (2)	0.0225 (2)	0.0305 (2)	−0.00170 (16)	0.00937 (14)	0.00038 (15)
C3	0.0365 (2)	0.0225 (2)	0.0305 (2)	−0.00170 (16)	0.00937 (14)	0.00038 (15)
C4	0.0585 (17)	0.0235 (14)	0.0460 (15)	−0.0007 (11)	0.0133 (13)	0.0053 (11)
C5	0.0587 (17)	0.0217 (13)	0.0508 (15)	0.0025 (11)	0.0088 (13)	−0.0033 (11)
C6	0.0369 (13)	0.0305 (13)	0.0391 (13)	0.0013 (10)	0.0024 (10)	−0.0061 (11)
C7	0.0505 (16)	0.0339 (15)	0.0503 (16)	0.0067 (12)	0.0107 (13)	−0.0111 (12)
C8	0.0533 (17)	0.0545 (19)	0.0459 (15)	0.0017 (14)	0.0149 (13)	−0.0147 (14)
C9	0.0548 (16)	0.0491 (18)	0.0363 (14)	−0.0036 (13)	0.0139 (12)	−0.0027 (12)
C10	0.0470 (14)	0.0310 (14)	0.0386 (14)	−0.0029 (11)	0.0063 (11)	−0.0023 (11)
C11	0.0308 (12)	0.0274 (12)	0.0322 (12)	−0.0015 (10)	0.0020 (9)	−0.0029 (10)
C12	0.0325 (12)	0.0227 (12)	0.0372 (13)	−0.0031 (9)	0.0112 (10)	−0.0017 (10)
C13	0.0453 (14)	0.0309 (13)	0.0379 (13)	−0.0020 (11)	0.0071 (11)	0.0032 (11)
C14	0.0634 (19)	0.0299 (15)	0.0612 (19)	0.0050 (13)	0.0162 (15)	0.0140 (14)
C15	0.069 (2)	0.0215 (14)	0.076 (2)	−0.0047 (13)	0.0246 (17)	−0.0030 (14)
C16	0.0535 (17)	0.0347 (15)	0.0563 (17)	−0.0109 (12)	0.0137 (13)	−0.0147 (13)
C17	0.0352 (13)	0.0311 (14)	0.0399 (13)	−0.0042 (10)	0.0086 (11)	−0.0050 (11)
C18	0.0590 (18)	0.0554 (19)	0.0512 (17)	−0.0095 (15)	−0.0150 (14)	0.0007 (15)
Cu1	0.0365 (2)	0.0225 (2)	0.0305 (2)	−0.00170 (16)	0.00937 (14)	0.00038 (15)
N1	0.0365 (2)	0.0225 (2)	0.0305 (2)	−0.00170 (16)	0.00937 (14)	0.00038 (15)
O1	0.0365 (2)	0.0225 (2)	0.0305 (2)	−0.00170 (16)	0.00937 (14)	0.00038 (15)

### Geometric parameters (Å, °)

**Table:**

C1—N1	1.303 (3)	C10—H10	0.9300
C1—C2	1.422 (3)	C12—C13	1.385 (3)
C1—H1	0.9300	C12—C17	1.398 (3)
C2—C3	1.420 (3)	C12—N1	1.432 (3)
C2—C11	1.452 (3)	C13—C14	1.382 (3)
C3—O1	1.292 (2)	C13—H13	0.9300
C3—C4	1.425 (3)	C14—C15	1.369 (4)
C4—C5	1.351 (3)	C14—H14	0.9300
C4—H4	0.9300	C15—C16	1.379 (4)
C5—C6	1.424 (3)	C15—H15	0.9300
C5—H5	0.9300	C16—C17	1.385 (3)
C6—C7	1.406 (3)	C16—H16	0.9300
C6—C11	1.417 (3)	C17—C18	1.501 (4)
C7—C8	1.361 (4)	C18—H18B	0.9600
C7—H7	0.9300	C18—H18C	0.9600
C8—C9	1.387 (4)	C18—H18A	0.9600
C8—H8	0.9300	Cu1—O1^i^	1.8881 (15)
C9—C10	1.374 (3)	Cu1—O1	1.8881 (15)
C9—H9	0.9300	Cu1—N1	1.9804 (17)
C10—C11	1.417 (3)	Cu1—N1^i^	1.9804 (17)
			
N1—C1—C2	127.2 (2)	C13—C12—N1	120.1 (2)
N1—C1—H1	116.4	C17—C12—N1	119.4 (2)
C2—C1—H1	116.4	C14—C13—C12	120.2 (2)
C3—C2—C1	119.9 (2)	C14—C13—H13	119.9
C3—C2—C11	119.2 (2)	C12—C13—H13	119.9
C1—C2—C11	120.33 (19)	C15—C14—C13	120.0 (3)
O1—C3—C2	124.4 (2)	C15—C14—H14	120.0
O1—C3—C4	116.62 (19)	C13—C14—H14	120.0
C2—C3—C4	119.0 (2)	C14—C15—C16	119.9 (3)
C5—C4—C3	121.4 (2)	C14—C15—H15	120.1
C5—C4—H4	119.3	C16—C15—H15	120.1
C3—C4—H4	119.3	C15—C16—C17	121.7 (3)
C4—C5—C6	122.0 (2)	C15—C16—H16	119.2
C4—C5—H5	119.0	C17—C16—H16	119.1
C6—C5—H5	119.0	C16—C17—C12	117.8 (2)
C7—C6—C11	120.2 (2)	C16—C17—C18	120.9 (2)
C7—C6—C5	121.3 (2)	C12—C17—C18	121.3 (2)
C11—C6—C5	118.5 (2)	C17—C18—H18B	109.5
C8—C7—C6	121.5 (2)	C17—C18—H18C	109.5
C8—C7—H7	119.2	H18B—C18—H18C	109.5
C6—C7—H7	119.2	C17—C18—H18A	109.5
C7—C8—C9	119.0 (2)	H18B—C18—H18A	109.5
C7—C8—H8	120.5	H18C—C18—H18A	109.5
C9—C8—H8	120.5	O1^i^—Cu1—O1	180.0
C10—C9—C8	121.4 (2)	O1^i^—Cu1—N1	90.07 (7)
C10—C9—H9	119.3	O1—Cu1—N1	89.93 (7)
C8—C9—H9	119.3	O1^i^—Cu1—N1^i^	89.93 (7)
C9—C10—C11	121.2 (2)	O1—Cu1—N1^i^	90.07 (7)
C9—C10—H10	119.4	N1—Cu1—N1^i^	180.00 (9)
C11—C10—H10	119.4	C1—N1—C12	117.21 (18)
C6—C11—C10	116.8 (2)	C1—N1—Cu1	122.60 (15)
C6—C11—C2	119.8 (2)	C12—N1—Cu1	119.73 (13)
C10—C11—C2	123.5 (2)	C3—O1—Cu1	127.52 (14)
C13—C12—C17	120.5 (2)		
			
N1—C1—C2—C3	−10.7 (4)	C17—C12—C13—C14	0.6 (4)
N1—C1—C2—C11	178.0 (2)	N1—C12—C13—C14	178.9 (2)
C1—C2—C3—O1	8.4 (3)	C12—C13—C14—C15	−0.3 (4)
C11—C2—C3—O1	179.9 (2)	C13—C14—C15—C16	0.4 (4)
C1—C2—C3—C4	−170.0 (2)	C14—C15—C16—C17	−0.8 (4)
C11—C2—C3—C4	1.5 (3)	C15—C16—C17—C12	1.0 (4)
O1—C3—C4—C5	−178.2 (2)	C15—C16—C17—C18	−179.9 (2)
C2—C3—C4—C5	0.3 (4)	C13—C12—C17—C16	−0.9 (3)
C3—C4—C5—C6	−0.5 (4)	N1—C12—C17—C16	−179.3 (2)
C4—C5—C6—C7	−179.7 (2)	C13—C12—C17—C18	−180.0 (2)
C4—C5—C6—C11	−1.1 (4)	N1—C12—C17—C18	1.7 (3)
C11—C6—C7—C8	−1.1 (4)	C2—C1—N1—C12	176.2 (2)
C5—C6—C7—C8	177.4 (3)	C2—C1—N1—Cu1	−11.6 (3)
C6—C7—C8—C9	−0.5 (4)	C13—C12—N1—C1	57.1 (3)
C7—C8—C9—C10	1.4 (4)	C17—C12—N1—C1	−124.5 (2)
C8—C9—C10—C11	−0.5 (4)	C13—C12—N1—Cu1	−115.3 (2)
C7—C6—C11—C10	1.9 (3)	C17—C12—N1—Cu1	63.1 (2)
C5—C6—C11—C10	−176.7 (2)	O1^i^—Cu1—N1—C1	−154.08 (18)
C7—C6—C11—C2	−178.5 (2)	O1—Cu1—N1—C1	25.92 (18)
C5—C6—C11—C2	2.9 (3)	O1^i^—Cu1—N1—C12	17.91 (16)
C9—C10—C11—C6	−1.1 (3)	O1—Cu1—N1—C12	−162.09 (16)
C9—C10—C11—C2	179.3 (2)	C2—C3—O1—Cu1	17.7 (3)
C3—C2—C11—C6	−3.1 (3)	C4—C3—O1—Cu1	−163.90 (16)
C1—C2—C11—C6	168.3 (2)	N1—Cu1—O1—C3	−29.77 (18)
C3—C2—C11—C10	176.4 (2)	N1^i^—Cu1—O1—C3	150.23 (18)
C1—C2—C11—C10	−12.2 (3)		

Symmetry codes: (i) −*x*, −*y*+1, −*z*.
